# Kinetics of Bacterial Adaptation, Growth, and Death at Didecyldimethylammonium Chloride sub-MIC Concentrations

**DOI:** 10.3389/fmicb.2022.758237

**Published:** 2022-04-07

**Authors:** Adrián Pedreira, José A. Vázquez, Míriam R. García

**Affiliations:** ^1^Biosystems and Bioprocess Engineering (Bio2Eng), Marine Research Institute-Spanish National Research Council (IIM-CSIC), Eduardo Cabello, Vigo, Spain; ^2^Group of Recycling and Valorization of Waste Materials (REVAL), Marine Research Institute-Spanish National Research Council (IIM-CSIC), Eduardo Cabello, Vigo, Spain

**Keywords:** dynamic modeling, disinfection, didecyldimethylammonium chloride (DDAC), *B. cereus*, *E. coli*, bacteriostatic, bactericidal, sub-MIC concentration

## Abstract

Minimum inhibitory concentration (MIC) and minimum bactericidal concentration (MBC) are standard indexes for determining disinfection effectiveness. Nevertheless, they are static values disregarding the kinetics at sub-MIC concentrations where adaptation, growth, stationary, and death phases can be observed. The understanding of these dynamic mechanisms is crucial to designing effective disinfection strategies. In this study, we studied the 48 h kinetics of *Bacillus cereus* and *Escherichia coli* cells exposed to sub-MIC concentrations of didecyldimethylammonium chloride (DDAC). Two mathematical models were employed to reproduce the experiments: the only-growth classical logistic model and a mechanistic model including growth and death dynamics. Although both models reproduce the lag, exponential and stationary phases, only the mechanistic model is able to reproduce the death phase and reveals the concentration dependence of the bactericidal/bacteriostatic activity of DDAC. This model could potentially be extended to study other antimicrobials and reproduce changes in optical density (OD) and colony-forming units (CFUs) with the same parameters and mechanisms of action.

## 1. Introduction

Designing effective disinfection strategies relies on understanding the mechanism of action (bacteriostatic, bactericidal, or both) and at which concentrations, using the well described and standardized minimum bactericidal concentration (MBC), minimum inhibitory concentration (MIC), or even others such as the non-inhibitory concentration (NIC) (Lambert and Pearson, 2000; EN-1276, 2009; ISO-20776-1, 2019).

Standard indexes of effective disinfectant concentrations are, however, endpoint static values, usually measured after 18 or 24 h of incubation and starting from a fixed inoculum without considering the inoculum effect and disregarding the bacterial kinetics (Mouton and Vinks, 2005; García and Cabo, 2018). Note that, even when effective supra-MBC treatments are applied, the kinetics of time-kill curves change with the disinfectant dose, as it has been widely analyzed in the literature using mathematical models (Gyrk and Finch, 1998; Peleg, 2021).

On the other hand, understanding the kinetics of ineffective disinfection treatments is also critical to prevent the emergence of resistance in bacteria. The non-volatile disinfectant may end on the environment at sub-MIC concentrations (Holah et al., 2002; García and Cabo, 2018; Ribič et al., 2020) with the corresponding emergence of resistant strains to the used disinfectant or even other antimicrobials as antibiotics (Capita and Alonso-Calleja, 2013; Nordholt et al., 2021), being nowadays especially relevant due to the overuse of disinfectants during the current SARS-CoV-2 pandemic (Pedreira et al., 2021). Moreover, even when supra-MIC treatments are applied, growth may be observed due to many practical problems as interfering substances or bacterial formation of biofilms (Simões et al., 2010; Araújo et al., 2013).

Mathematical models analyzing the sub-MIC treatments, nevertheless, are only focused on non-disinfectant antimicrobials such as antibiotics or toxic substances or particles (Liu et al., 2005; Vázquez et al., 2011; Theophel et al., 2014) and are only-growth models disregarding any decrease in bacterial population numbers (Chatterjee et al., 2015). Models combining net growth and death rates are needed since sub-MIC kinetics curves show lag, exponential, stationary, and death phases and those models can help to determine if a disinfectant works by increasing the lag phase, decreasing the growth rate (bacteriostatic), increasing the death rate (bactericidal), or a combination of both mechanisms depending on its concentration.

The main urge to model sub-MIC kinetics is for quaternary ammonium compounds (QACs), non-volatile disinfectants with the risk to promote resistance (Kampf, 2018a). They are cost-effective disinfectants with detergency properties, and thus, they can be used as single-stage cleaning and disinfection agents in low soiling conditions. QACs are widely employed in industry, household a and even cosmetics products as detergents, emulsifiers, softeners, disinfectants, or floating agents (Zhang et al., 2015). This family comprises several amphiphilic cationic surfactants with a general structure of N+R1R2R3R4X-, where R represents a hydrogen atom, an alkyl group, or other functional groups, and X represents an anion (Buffet-Bataillon et al., 2012).

Didecyldimethylammonium chloride (DDAC) is a commonly employed QAC formed by two alkyl chains each comprising of 10 (C10, didecyl) carbon atoms. UE legislation also includes under the name of DDAC such mixtures comprising C8 (octyl), C10, and C12 (dodecyl) chains, with at least 90% of C10 chains (EURL-SRM, 2016). DDAC is commonly found in the formulation of healthcare products and household cleaners/sanitizers as well as in the disinfection of surfaces and equipment in the food and feed areas both alone and in mixtures with other detergents (Lim and Chung, 2014; EURL-SRM, 2016). DDAC is especially common in the dairy industry, where it is widely used for disinfection of all kinds of surfaces on milking equipment, milk storage tanks, and machines and even in the disinfection of udders in order to prevent mastitis (EURL-SRM, 2016). When employed correctly, DDAC is considered safe to operatives and consumers and causes minimal corrosion of common materials, particularly compared to oxidative disinfectants.

Didecyldimethylammonium chloride is antimicrobial acting on the bacterial membranes and showing bacteriostatic and bactericide activity depending on its concentration and the growth phase of the population (Yoshimatsu and Hiyama, 2007; Kampf, 2018a). The MIC values for DDAC vary among species and isolates but its application at the recommended dosage in commercial disinfectants seems to be not always enough to inactivate all types of pathogens (Ramzi et al., 2020). In general, most bacteria isolated from food have MIC values ranging from 0.5 to 6.0 mg L-1 (Kampf, 2018a). However, MIC values up to 1,024 mg L-1 for DDAC and others QACs have been reported in bacteria isolated from retail meats in China (Zhang et al., 2016) and the USA (Zou et al., 2014).

Large DDAC MIC values could be related to the ability of bacteria to acquire resistance when exposed to sub-MIC concentrations, as have been observed in *E. coli* , with an increase of 1.5 to 3-fold in MIC value (Kampf, 2018a). Moreover, DDAC could promote cross-resistance to other disinfectants and antibiotics (Langsrud et al., 2003; Walsh et al., 2003; Soumet et al., 2016; Kampf, 2018b). The presence of these sub-MIC disinfectant concentrations on surfaces can be explained as a consequence of an incorrect calculation of the disinfectant work concentration, the employ of expired or inappropriate stored substances (with the consequent decrease in efficiency), the lack of a successful pre-disinfection cleaning to remove organic matter (responsible of inactivation of disinfectant substances) or an irregular spreading (Capita et al., 2019).

The aim of this study is to motivate the need for new mechanistic models to study disinfection at sub-MIC treatments and demonstrate the insight gained by this analysis. The case study is the evaluation of the effect of DDAC on the growth of the common foodborne pathogens *E. coli* and *B. cereus* (LeeNari et al., 2014) using measurements of optical density (OD) and colony-forming units (CFUs/ml). Two models are compared, the classical logistic only-growth model, where the DDAC effect is described using the Weibull functions, and a new mechanistic model that describes explicitly the mechanisms of adaptation, growth, and death using the Hill equations for the disinfectant effect. The mechanistic model shows a better goodness-of-fit for OD growth and is analyzed and extended to reproduce growth with both usual measurements (OD and CFUs/ml) and to understand DDAC treatment.

## 2. Materials and Methods

### 2.1. Bacterial Strains and Culture Conditions

Nonpathogenic surrogates strains of *B. cereus* (CECT 495) and *E. coli* (CECT 102) were purchased to Colección Española de Cultivos Tipo (CECT, Universidad de Valencia, Spain). Both *B. cereus* and *E. coli* are common foodborne pathogens and were chosen by their differences at cell wall level, respectively Gram-positive and Gram-negative. Stock cultures were kept at -80°C in culture media supplemented with 25% (v/v) glycerol.

The culture media selected for the dose-response assays was meat-peptone broth (MPB) containing 5 gL^−1^ meat extract (Scharlau SL, Barcelona, Spain), 10 gL^−1^ neopeptone (BactoTM. BD Biosciences, Franklin Lakes, NJ, USA), and 5 gL^−1^ NaCl (Emsure®, Merck KGaA, Darmstadt, Germany) in distilled water. DDAC (98 % purity; ABCR GmbH & Co KG, Karlsruhe, Germany) was added to the media to obtain the different desired concentrations for sub-MIC treatments. The pH was adjusted to 7.2 and the media was finally sterilized by autoclaving (121°C/15 min). Dose-response assays were performed in 300 mL Erlenmeyer flasks containing 180 mL of MPB. Flasks were inoculated with 900 μL from a 21 h culture and incubated in an orbital shaker (200 rpm) at 30°C ( *B. cereus* ) or 37°C ( *E. coli* ). A previous screening was carried out to know the range of DDAC concentrations with partial inhibitory effect under our specific experimental conditions. The resulting DDAC concentrations selected were 0.5, 0.75, 1.00, 1.50, 2.00, and 3.00 mg L-1 for *E. coli* and 0.05, 0.07, 0.25, 0.80, 1.00, and 1.50 mg L-1 for *B. cereus* . Each DDAC concentration was tested by duplicate. At predetermined incubation times (0, 2, 4, 8, 12, 16, 20, 24, 30, 36, and 48 h), samples from each flask were taken and properly diluted in peptone-buffered water for OD determination at 700 nm. For comparative purposes, 1 mL samples from each flask were several-fold diluted in peptone-buffered solution and 0.1 mL aliquots were plated by duplicate on agarized MPB (2% w/v agar), resulting in a total of four plates per DDAC concentration tested for each strain. Plates were incubated at previously indicated temperatures for each strain and manually counted after 24–48 h of incubation.

### 2.2. Mathematical Modeling of Bacterial Dynamics at sub-MIC Concentrations

Different types of models have been studied in this study. First, we compared the performance of two types of models to reproduce OD growth and inhibition by DDAC: the classical logistic model with disinfectant inhibition following the Weibull functions and a dynamic model based on mechanisms of adaptation, growth, inhibition, and death. Second, we extend the mechanistic model to account for CFUs growth. Finally, we present the numerical methods used for the simulation, parameter estimation, and model analysis.

#### 2.2.1. The Logistic Model With the Weibull Functions to Describe the Disinfectant Inhibition

The first model applied for the description of DDAC effect on bacterial growth measured with OD was a bivariate equation based on the combination of the Weibull function as biocide-concentration model modifying the most relevant kinetic-parameters of the reparametrized logistic equation used for the bacterial growth description (Rial et al., 2011; Vázquez et al., 2011):


(1) $$\begin{array}{l} OD=X=\frac{X_{m}}{1+\exp\left(2+\frac{4V_{m}}{X_{m}}\left(\lambda -t\right)\right)} \end{array}$$

being *X*, the OD (absorbance at 700 nm, OD700) dependent on three functions of DDAC concentration (*C*) describing the maximum OD (*X*_*m*_), the maximum growth rate (*V*_*m*_), and the lag phase (λ). These functions are assumed that comply with the Weibull functions as follows:


(2) $$\begin{array}{l} X_{m}=X_{m}^{0}\left[1-K_{x}\left(1-\exp\left\{-\ln\left(2\right){\left(\frac{C}{m_{x}}\right)}^{a_{x}}\right\}\right)\right] \end{array}$$

(3) $$\begin{array}{l} V_{m}=V_{m}^{0}\left[1-K_{v}\left(1-\exp\left\{-\ln\left(2\right){\left(\frac{C}{m_{v}}\right)}^{a_{v}}\right\}\right)\right] \end{array}$$

(4) $$\begin{array}{l} \lambda =\lambda^{0}\left[1-K_{\lambda }\left(1-\exp\left\{-\ln\left(2\right){\left(\frac{C}{m_{\lambda }}\right)}^{a_{\lambda }}\right\}\right)\right] \end{array}$$

Parameters meaning and units are summarized in Table 1.

**Table 1:** Variables and parameters used for the logistic model.

**Variables**		**Units**
*t*	Time	h
*X*	Bacterial concentration measured with absorbance at 700 nm.	AU
*X*_*m*_	Maximum bacterial load	AU
*V*_*m*_	Maximum growth rate	AU h-1
λ	Lag phase	h
*C*	Concentration of disinfectant	mg L-1
**Parameters**		
$X_{m}^{0}$	Maximum bacterial load without disinfectant	AU
*K*_*x*_	Maximum response affecting on *X*_*m*_	1
*m*_*x*_	Disinfectant corresponding to the semi-maximum response affecting on *X*_*m*_	mg L-1
*a*_*x*_	Shape parameter affecting on *X*_*m*_	1
$V_{m}^{0}$	Maximum growth rate without disinfectant	AU h-1
*K*_*v*_	Maximum response affecting on *V*_*m*_	1
*m*_*v*_	Disinfectant corresponding to the semi-maximum response affecting on *V*_*m*_	mg L-1
*a*_*v*_	Shape parameter affecting on *V*_*m*_	1
λ^0^	Lag phase without disinfectant	h
*K*_λ_	Maximum response affecting on λ	1
*m*_λ_	Disinfectant corresponding to the semi-maximum response affecting on λ	mg L-1
*a*_λ_	Shape parameter affecting on λ	1

*AU, Absorbance units*.

#### 2.2.2. The Mechanistic Model With the Hill Equations to Describe the Disinfectant Effect

As an alternative to the classical logistic model with the Weibull functions, we derived a model based on cell adaptation, substrate-based growth with inhibition by cell density and death. The idea can be outlined using the following biochemical reactions:


(5) $$\begin{array}{ll} \text{Adaptation of latent cells to adapted cells} & X_{l}\overset{k_{a}}{\underset{\;}{\to }}X \end{array}$$

(6) $$\begin{array}{ll} \text{ Substrate-based-growth} & X+\left(1/Y_{S}\right)S\overset{\left(\frac{k_{g}}{k_{i}+X}\right)}{\underset{\;}{\to }}2X\\ \text{with inhibition by cell density} \end{array}$$

(7) $$\begin{array}{ll} \text{Death of latent cells} & X_{l}\overset{k_{d}}{\underset{\;}{\to }}0 \end{array}$$

(8) $$\begin{array}{ll} \text{Death of adapted cells} & X\overset{k_{d}}{\underset{\;}{\to }}0 \end{array}$$

where *X*_*l*_ and *X* are, respectively, the OD of latent and adapted cells and *S* is the available substrate that is consumed (with yield coefficient *Y*) when adapted cells divide. The mass action was assumed for all the biochemical reactions (i.e., constant rates, *k*_*a*_ and *k*_*d*_ for adaptation and death, respectively, multiplied for the reactants) with exception of the growth rate. In this reaction, we included the inhibition by cell density using Michaelis–Menten kinetics.

The mass balance of the reactions results on the following equations:


(9) $$\begin{array}{lll} \frac{\text{d}X_{l}}{\text{d}t} & = & -k_{a}X_{l}-k_{d}X_{l} \end{array}$$

(10) $$\begin{array}{lll} \frac{\text{d}X}{\text{d}t} & = & k_{a}X_{l}+\left(\frac{k_{g}}{k_{i}+X}\right)SX-k_{d}X \end{array}$$

(11) $$\begin{array}{lll} \frac{\text{d}S}{\text{d}t} & = & -Y_{S}\left(\frac{k_{g}}{k_{i}+X}\right)SX \end{array}$$

(12) $$\begin{array}{lll} OD & = & \left(X_{l}+X\right)+\alpha X_{d} \end{array}$$

(13) $$\begin{array}{lll} CFUs & = & \beta 10^{10}\left(X_{l}+X\right) \end{array}$$

consisting of three ordinary differential equations (ODEs) and two types of measurable variables:

- *Optical density*: sum of the contribution of the latent (*X*_*l*_) and adapted cells (*X*) plus non-lysed death cells as assumed by Haque et al. (2017). Note that dead cells can be calculated as *X*_*d*_ = *X*_*l*_(*t* = 0)−(*X*_*l*_+*X*) and that the percentage of non-lysed cells is assumed constant and represented by α∈[0, 1].
- *Colony-forming units*: sum of alive cells (latent or adapted) multiplied by a scale factor.

To understand the effect of the disinfectant on the different mechanisms, we added dependence of the disinfectant concentration using the Hill equations:


(14) $$\begin{array}{lll} k_{g} & = & k_{g}^{0}\frac{{IC_{50,g}}^{\gamma_{g}}}{C^{\gamma_{g}}+{IC_{50,g}}^{\gamma_{g}}} \end{array}$$

(15) $$\begin{array}{lll} k_{a} & = & k_{a}^{0}\frac{{IC_{50,a}}^{\gamma_{a}}}{C^{\gamma_{a}}+{IC_{50,a}}^{\gamma_{a}}} \end{array}$$

(16) $$\begin{array}{lll} k_{d} & = & k_{d}^{0}+k_{d}^{*}\frac{C^{\gamma_{d}}}{C^{\gamma_{d}}+{EC_{50,d}}^{\gamma_{d}}} \end{array}$$

where decreasing Hill functions are assumed to model inhibition of growth and adaptation rates with increasing disinfectant concentration (decreasing dose-response curves), and an increasing Hill function is assumed to describe the increase of the death rate with the disinfectant (increasing dose-response curve) (Mouton and Vinks, 2005; Santillán, 2008). In these equations, γ is the Hill coefficient shaping the effect of the disinfectant (higher values model sharp functions, similar to step functions with only two possible values, and low values describe an almost constant function except for a jump at zero and around 1 simulates a sigmoid curve) and *IC*_50_ and *EC*_50_ are the half maximal inhibitory/effective concentration (disinfectant concentrations at which 50% of the maximum effect is obtained). Note that without disinfectant the growth and adaptation velocities correspond with $k_{g}^{0}$ and $k_{a}^{0}$. In absence of disinfectant, we allow death at $k_{d}^{0}$ to reproduce the final death phase, whereas $k_{d}^{*}$ scales the effect of the disinfectant. The variables and parameters are summarized in Table 2.

**Table 2:** Variables, initial conditions, and parameters used for the mechanistic model.

**Variables**		**Units**
*t*	Time	h
*X*_*l*_	Optical density of latent cells	AU
*X*	Optical density of adapted cells	AU
*S* ^†^	Substrate for growth (availability of resources)	1
*C*	Concentration of disinfectant	mg L-1
*K*_*a*_ = *k*_*a*_	Adaptative specific rate	h-1
$K_{g}=\frac{k_{g}}{k_{i}+X}$	Growth specific rate	h-1
*K*_*d*_ = *k*_*d*_	Death specific rate	h-1
Initial conditions		
*X*_*l*_(*t* = 0) = *OD*(*t* = 0)	Initial density of latent cells	AU
*X*(*t* = 0) = 0	Initial density of adapted cells	AU
*S*(*t* = 0) = 1	Initial substrate density, normalized to 1	1
**Parameters**		
$k_{a}^{0}$	Adaptation rate without disinfectant	h-1
$k_{g}^{0}$	Growth rate without disinfectant	AU h-1
$k_{d}^{0}$	Death rate without disinfectant	h-1
$k_{d}^{*}$	Scaling of disinfectant effect on death	h-1
*k*_*i*_	Inhibition constant due to cell density	AU
*IC*_50, *a*_	Half maximal inhibitory concentration of adaptation rate	mg L-1
*IC*_50, *g*_	Half maximal inhibitory concentration of growth rate	mg L-1
*EC*_50, *d*_	Half maximal effective concentration on death	mg L-1
γ_*a*_	Effect shape of disinfectant over adaptation rate	1
γ_*g*_	Effect shape of disinfectant over growth rate	1
γ_*d*_	Effect shape of disinfectant over death rate	1
*Y*_*S*_	Yield coefficient	1/AU
α	Contribution of death cells to OD	1
β	Scaling factor from *OD* to *CFUs*	CFUs /AU mL

*AU, Absorbance units. ^†^ Normalized substrate concentration is considered as the initial substrate is not identifiable*.

### 2.3. Numerical Methods

For the model simulation, calibration (estimation of unknown parameters), and analysis, we use numerical methods implemented in AMIGO2 (Advanced Model Identification using Global Optimization) software, a multi-platform toolbox implemented in Matlab (Balsa-Canto et al., 2016b). The code, with all the selected options for simulation and optimization, is freely available at https://doi.org/10.5281/zenodo.5167910.

For the model calibration, we have selected the maximization of the log-likelihood function, equivalent under common assumptions [refer to Balsa-Canto et al. (2016a) for details] to minimize the least-squares function (squares of the residuals) divided by the standard deviation. In this study, we calculate the standard deviation for each time-point from the experimental replicates (refer to calculated standard deviations for each time-point in Supplementary Figure S1 and calculate the mean for each bacteria ( *B. cereus* , *E. coli* ) and type of data (CFUs, OD). Therefore, the objective is to find the parameters that minimize:


(17) $$\begin{array}{l} J=\frac{1}{\sigma^{2}}\overset{n_{exp}}{\underset{j=1}{\sum }}\overset{n_{t}}{\underset{i=1}{\sum }}{\left(Y_{i,j}-{Ŷ}_{i,j}\right)}^{2},\text{with }\sigma =\bar{\sigma_{ij}} \end{array}$$

where *n*_*exp*_ and *n*_*t*_ are the number of experiments (seven including the control without disinfectant for each bacterial strain) and the number of time data points, respectively, and being *Y*, σ, and Ŷ the data, standard deviation, and model predictions, respectively. Bounds considered for the parameters were [0, 15] with the exception of α∈[0, 1] and β∈[0, 100].

The confidence intervals for the parameters were estimated by $\pm t_{\alpha /2}^{\gamma }\sqrt{C_{ii}}$, where *C*_*ii*_ are the diagonal elements of the confidence matrix, $t_{\alpha /2}^{\gamma }$ is given by Student's *t*-distribution with γ the number of degrees of freedom and (1−α)100% selected to 95%. The Cramér-Rao inequality was employed to compute a bound for the confidence matrix using the Fisher information matrix (Balsa-Canto et al., 2016a).

To compare the performance among models, we employed the following well-known indexes: the adjusted *R*^2^, the corrected Akaike information criterion (AICc), and the Bayesian information criterion (BIC). The three indexes are a function of the error existing between model and experimental data, penalizing the number of parameters to be estimated. Indexes with lower numbers imply better performance.

## 3. Results and Discussion

### 3.1. Modeling Growth Measured With OD

In this study, we compared a classical model (logistic growth with inhibition using the Weibull functions) with a model derived from mechanisms (inspired by biochemical reactions of adaptation, substrate-based growth with inhibition by cell density and death).

As explained in section 2.2.1, the classical model describes the effect of DDAC on bacterial growth based on the combination of the Weibull function as biocide-concentration model modifying the most relevant kinetic-parameters of the reparametrized logistic equation used for the bacterial growth description. We should mention that the derivative form of this model was used to allow its implementation in the AMIGO2 software, refer to https://doi.org/10.5281/zenodo.5167910 for details.

On the other hand, the model derived in this study assumes the following mechanisms (refer to section 2.2.2 for mathematical details of the model):

- Initially, cells are in a latent state (*X*_*l*_) without the potential to divide. The adaptation of latent cells can be interpreted in terms of the following biochemical reaction:

$$X_{l}\overset{k_{a}X_{l}}{\underset{\;}{\to }}X$$

where the specific adaptation rate (or rate per capita) is a Hill-decreasing-function of the disinfectant concentration *k*_*a*_(*C*), as described later, and the total rate follows the mass action law (*K*_*a*_ = *k*_*a*_*X*).

- Adapted cells (*X*) grow if there is disponibility of nutrients (substrate *S*) and this growth is inhibited by cell density using Michaelis–Menten kinetics. The biochemical representation is as follows:

$$X+\left(1/Y_{S}\right)S\overset{\frac{k_{g}}{k_{i}+X}SX}{\underset{\;}{\to }}2X$$

where 1/*Y*_*S*_ is the yield coefficient, the specific growth rate is again a Hill-decreasing-function of disinfectant *k*_*g*_(*C*) and *k*_*i*_ is the coefficient regulating the inhibition due to cell density (with low values for strong inhibition by large cell numbers). Note that this inhibition encodes different mechanisms like the inhibition for the production of waste in a cell cycle. This biochemical reaction describes both the exponential growth phase and the stationary phase.

- Latent and adapted cells may die:

$$X_{l}\overset{k_{d}X_{l}}{\underset{\;}{\to }}0$$

$$X\overset{k_{d}X}{\underset{\;}{\to }}0$$

Death occurs with a specific rate dependent on a Hill-increasing function on the concentration of disinfectant *k*_*d*_(*C*). Note that death may be active during the whole dynamics and determines the death phase when this rate surpasses the growth rate (negative cell growth).

- Cellular adaptation, growth, and death are assumed to depend on DDAC concentration. As adaptation and growth rates commonly decrease with disinfectant concentration, we selected a decreasing dose-response curve, whereas for death an increasing dose-response curve was assumed. For the shape of the dose-response, we employed the Hill Equations (14–16): standard functions on pharmacodynamics selected due to their flexibility and their mechanistic interpretation, providing estimations of half maximal inhibitory/effective concentration of the disinfectant over the different mechanisms (Regoes et al., 2004).
- Optical density includes the direct contribution of the latent and adapted viable cells plus some contribution [to be estimated, as previously assumed in Haque et al. (2017)] of the dead cells (including those viable but not cultivable cells undetected by plating). Mathematically, this is expressed as follows where dead cells can be calculated as the initial total cells [only latent cells *X*_*l*_(*t* = 0)] minus latent and adapted cells at a given time:

$$OD=\left(X_{l}+X\right)+\alpha X_{d}=\left(X_{l}+X\right)+\alpha \left[X_{l}\left(t=0\right)-\left(X_{l}+X\right)\right]$$

Under these assumptions, we derived a model consisting of a set of three ODEs plus an algebraic equation defining the measured variable *OD* (refer to section 2.2.2). We should mention that for its implementation in the AMIGO2 toolbox, the derivative equation of the *OD* function was implemented. Refer to code for details at https://doi.org/10.5281/zenodo.5167910.

**Figure 1:**
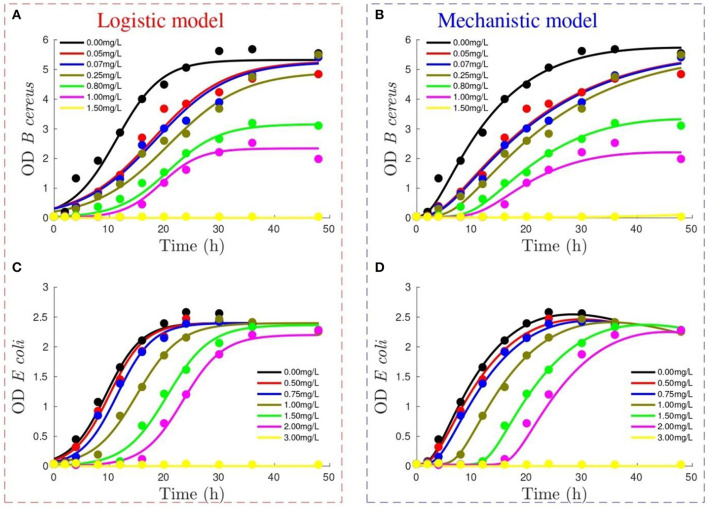
Performance of logistic model (figures on the left) and mechanistic model (figures on the right) to reproduce optical density (OD) growth of *Bacillus cereus* **(A,B)** and *Escherichia coli* **(C,D)** at different Didecyldimethylammonium chloride (DDAC) concentrations (refer to legend). Lines show model output, whereas experimental data are represented by dots.

Figure 1 depicts the performance of the logistic and mechanistic model to reproduce the growth of *B. cereus* and *E. coli* measured using OD. Both models reproduce the data trends, with the main difference being the ability of the mechanistic model to describe the smooth transition to the stationary phase in *B. cereus* (Figure 1B) and the initial death phase for *E. coli* (Figure 1D). Moreover, the mechanistic model starts from the experimental initial conditions, whereas the logistic model allows flexibility for this condition.

Table 3 shows three standard indexes to measure the model's ability to reproduce the data penalizing overparametrisation: the adjusted *R*^2^, the corrected AICc, and the BIC. All criteria show a lower value (despite penalizing the models with many degrees of freedom) for the mechanistic model, where 13 parameters were estimated instead of the 12 estimation parameters for the logistic model.

**Table 3:** Performance of both models to reproduce the data (measured in terms of the Adjusted R^2^, AICc, BIC) and estimated parameters.

	***B. cereus***		***E. coli***	
	**Logistic model**	**Mechanistic model**	**Logistic model**	**Mechanistic model**
Adj. R^2^	0.97	0.98	0.99	1.00
AICc	3,116.59	1,831.19	16,229.24	7,152.58
BIC	3,141.37	1,857.59	16,253.38	7,178.28
Parameters	$X_{m}^{0}$ = 5.3	$k_{a}^{0}$ = 15	$X_{m}^{0}$ = 2.4	$k_{a}^{0}$ = 0.35
	*K*_*x*_ = 14	$k_{g}^{0}$ = 0.59	*K*_*x*_=11	$k_{g}^{0}$ = 0.33
	*m*_*x*_ = 6.9	$k_{d}^{0}$ = 0.003	*m*_*x*_ = 4.2	$k_{d}^{0}$ = 0.023
	*a*_*x*_ = 1.5	$k_{d}^{*}$ = 15	*a*_*x*_ = 6.2	$k_{d}^{*}$ = 12
	$V_{m}^{0}$ = 0.33	*k*_*i*_ = 0.51	$V_{m}^{0}$ = 0.19	*k*_*i*_ = 0.24
	*K*_*v*_ = 1.1	*IC*_50, *a*_ = 0.021	*K*_*v*_ = 0.19	*IC*_50, *a*_ = 0.71
	*m*_*v*_ = 5	*IC*_50, *g*_ = 15	*m*_*v*_=0.91	*IC*_50, *g*_ = 15
	*a*_*v*_ = 0.11	*EC*_50, *d*_ = 3.1	*a*_*v*_ = 15	*EC*_50, *d*_ = 3.7
	λ^0^ = 3.2	γ_*a*_ = 1.9	λ^0^ = 3	γ_*a*_ = 15
	*K*_λ_ = 10	γ_*g*_ = 0.076	*K*_λ_ = 4.7	γ_*g*_ = 0.68
	*m*_λ_ = 3.2	γ_*d*_ = 4.7	*m*_λ_ = 1.3	γ_*d*_ = 14
	*a*_λ_ = 0.65	*Y*_*S*_ = 0.16	*a*_λ_ = 2.9	*Y*_*S*_ = 0.22
		α = 0.32		α = 0.0001

The mechanistic model was selected for the rest of the study due to a better goodness-of-fit and its mechanistic insight. We want to stress that although both models show good performance, which was only slightly lower for the classical model, the insight from the mechanistic model was determinant to make the decision. Mechanistic models allow a deeper study of the process and (as shown in the next section) can be extended to account also for CFUs by adding only one extra parameter.

### 3.2. Extending the Mechanistic Model to Reproduce Growth Measured With CFUs

Both OD and CFUs are related standard methods to measure bacterial concentration dynamics but differ in their interpretation. OD is a fast method but is affected by many factors, including dead cells in the medium with intact cell wall (Stevenson et al., 2016), making estimations of alive cells unreliable, whereas CFUs provides reliable estimations of viable cells, but it is experimentally time-consuming. Estimations using only CFUs were tested with both logistic and mechanistic models (data not shown), with better estimations for the mechanistic model for including the death phase observed in the data.

We extended the mechanistic model derived in this study to explain both, OD and CFUs data. Let us recall here the OD variable:


$$OD=\left(X_{l}+X\right)+\alpha X_{d}$$

being a function of latent, adapted, and dead cells. For the modeling of CFUs, we consider only alive cells (either latent or adapted):


$$CFUs=\beta 10^{10}\left(X_{l}+X\right)$$

with β10^10^ a scaling factor transforming *OD* to *CFUs*.

**Figure 2:**
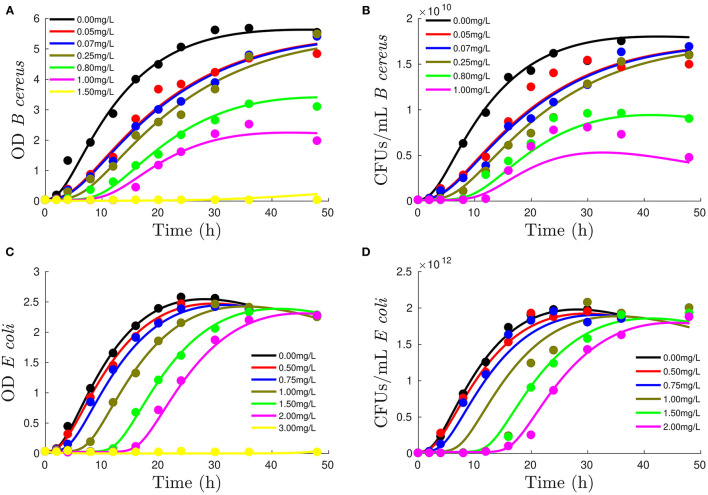
Performance of mechanistic model to reproduce growth measured with OD [figures on the left, **(A,C)**] and colony-forming units (CFUs) [figures on the right column, **(B,D)**] for *B. cereus* [first row, **(A,B)**] and *E. coli* [second row, **(C,D)**] at different DDAC concentrations (refer to legend). Lines show model output, whereas experimental data are represented by dots.

Figure 2 shows how assumed mechanisms are sufficient to reproduce OD and CFUs using the same adaptation, growth, and death parameters. The mechanistic model has 14 parameters: 12 common parameters plus α, defining contribution of dead cells to OD, and β10^10^, which represents the difference between OD (usually one order of magnitude) and CFUs (varying from 10 orders of magnitude for *B. cereus* and 12 for *E. coli* ).

Table 4 shows the model performance in terms of adjusted *R*^2^, AICc, and BIC. Note that now the model has been challenged to reproduce both OD and CFUs with the same parameters and, although indexes are acceptable for the measured experimental errors, are worse than when reproducing only OD. To further analyze that the performance is satisfactory, we have included the confidence intervals of the estimated parameters, where the data uncertainty is also considered (refer to Supplementary Figure S1).

**Table 4:** Results (performance indexes and estimated parameters with their confidence intervals) for the extended mechanistic model to account for both *OD* and *CFUs* growth with DDAC.

	***B. cereus***	***E. coli***
Adj. R^2^	0.96	0.96
AICc	3,147.29	21,026.27
BIC	3,188.28	21,066.64
Parameters ± CI	$k_{a}^{0}$ = 15 ± 63	$k_{a}^{0}$ = 0.68 ± 0.13
	$k_{g}^{0}$ = 0.6 ± 0.025	$k_{g}^{0}$ = 0.34 ± 0.0059
	$k_{d}^{0}$ = 0.0035 ± 0.0016	$k_{d}^{0}$ = 0.022 ± 0.0015
	$k_{d}^{*}$ = 13 ± 62	$k_{d}^{*}$ = 15 ± 2.2
	*k*_*i*_ = 0.54 ± 0.057	*k*_*i*_ = 0.29 ± 0.016
	*IC*_50, *a*_ = 0.023 ± 0.053	*IC*_50, *a*_ = 0.65 ± 0.0068
	*IC*_50, *g*_ = 14 ± 24	*IC*_50, *g*_ = 15 ± 2.2
	*EC*_50, *d*_ = 3 ± 3.3	*EC*_50, *d*_ = 3.8 ± 0.05
	γ_*a*_ = 1.8 ± 0.22	γ_*a*_ = 12 ± 0.46
	γ_*g*_ = 0.078 ± 0.024	γ_*g*_ = 0.71 ± 0.039
	γ_*d*_ = 4.8 ± 0.25	γ_*d*_ = 15 ± 0.61
	*Y*_*S*_ = 0.16 ± 0.0067	*Y*_*S*_ = 0.23 ± 0.0082
	α = 0.28 ± 0.027	α = 6.9e-07 ± 0.013
	β = 0.33 ± 0.0045	β = 78 ± 0.47

**Figure 3:**
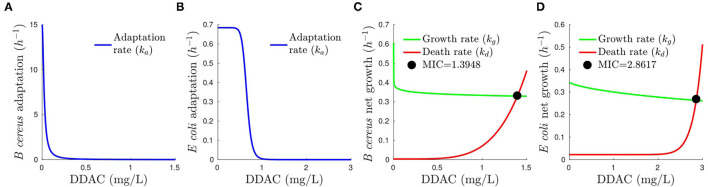
Dependence of adaptation, growth (under the assumptions of non-inhibition by substrate availability and cell density), and death specific rates on DDAC. The bacteriostatic action is seen in the decrease of growth rate (kg) and the bactericidal in the increase of death rate (kd) as a function of DDAC concentration for *B. cereus* **(A)** and *E. coli* **(B)**. The intersection of growth and death rates gives the minimum concentration of DDAC for which net growth is zero (Coates et al., 2018) for *B. cereus* **(C)** and *E. coli* **(D)**.

The graphical picture of how adaptation, growth, and death-specific rates change with DDAC is shown in Figure 3. Note that growth-specific rate is inhibited by limiting substrates and large cell density. To simplify the figure, we plotted *k*_*g*_ assuming that resources are not limiting (*S* is almost constant) and inhibition by cell density is not relevant, and therefore, *K*_*g*_≈*k*_*g*_.

### 3.3. Discussion

In this study, we derived a new mechanistic model able to represent the different phases (adaptation, growth, stationary, and death) at sub-MIC concentrations of DDAC. The logistic model, the standard model used to quantify growth inhibition with bacteriostatic antimicrobials, was unable to reproduce the last death phase of the growth curves. This limitation was previously detected when modeling the inhibition of *E. coli* and *Staphylococcus aureus* due to silver nanoparticles (Chatterjee et al., 2015). As with sub-MIC DDAC concentrations, nanoparticles are mostly bacteriostatic but growth curves show a decline at long times.

The derived model was calibrated from and did reproduce the two most common measurements of bacterial population numbers, OD and CFUs, using the same parameters for adaptation, growth, and death rate. Results show that *E. coli* dead cells do not affect OD, probably due to severe cell lysis and their not appreciable effect in OD measurement (Stevenson et al., 2016). However, 32% of *B. cereus* dead cells contribute to OD. Two hypotheses can explain this estimation: either this contribution is due to viable but nonculturable cells or either some cells are non-lysed since *B. cereus* has more resistance to lysis than *E. coli* (Fykse et al., 2003).

The analysis of the developed model confirms the literature observation that DDAC is bacteriostatic and bactericidal depending on its concentration and the growth phase of the microbial population (Yoshimatsu and Hiyama, 2007; Kampf, 2018a). The growth rate is affected in both *B. cereus* and *E. coli* . In *B. cereus* the growth decreases sharply only by adding low concentrations of DDAC and remains almost constant afterward (γ_*g*_ close to zero), as can be seen in Figure 3, being, therefore, *IC*_50, *g*_ irrelevant and with large uncertainty. Although Hill functions are very uncertain, and not useful to infer exactly at which concentration is this sharp decrease, the growth rate should be at least 0.6 h^−1^ when DDAC = 0 mg L-1and 0.4 h^−1^ for DDAC = 0.5 mg L-1 (the DDAC concentrations experimentally tested) to fit the data. In *E. coli* , growth decreases smoothly when DDAC increases (γ_*g*_ = 0.078 and *IC*_50, *g*_ = 14). This can be also observed in Figures 3C,D for *B. cereus* and *E. coli* , respectively. On the other hand, the bactericidal effect is also detected for both *B. cereus* and *E. coli* despite being the estimated dying rates the most uncertain model kinetics (as evidenced by the parameter confidence intervals). Whereas for *B. cereus* the bactericidal effect increases smoothly with DDAC concentration (Figure 3C), this behavior is more abrupt for *E. coli* .

The model also allows the study of the DDAC changes in the lag phase, a mechanism that is not considered when simply classifying a disinfectant as bacteriostatic or bactericidal. Adaptation is fast in *B. cereus* (small lag phase) and slow in *E. coli* . The model captures this behavior using larger values of *k*_*a*_ for *B. cereus* (maximum allowed value $k_{a}^{0}=15$) than for *E. coli* ($k_{a}^{0}=0.35$). Note that large confidence intervals are reported for *B. cereus* adaptation rate as faster adaptation rates result in the same dynamics where adaptation is complete within minutes or seconds at *c* = 0. Despite both strains showing the different magnitude of adaptation, the change of adaptation with DDAC concentration (Figures 3A,B) shows a similar trend. Whereas for both adaptation is high without DDAC, when DDAC concentration increases there is a deep decrease of adaptation, therefore, showing longer lag phases at high DDAC concentrations.

The analysis of the lag phase with DDAC concentration is particularly relevant to understanding the implications of MIC values and how they depend on the growth phase (Yoshimatsu and Hiyama, 2007; Kampf, 2018a). Lack of growth after 24 h could be due to different mechanisms: either an extremely large lag phase or that the death rate is equal or larger than the growth rate [zero or negative net growth rate, also commented by Mouton and Vinks (2005) the stationary concentration]. Assuming no effect of the limiting substrate and MIC representing the concentration of zero growth rate (Coates et al., 2018), the model can be used to calculate the MIC using the intersection of growth and death rate (*k*_*g*_ and *k*_*d*_). As shown in Figure 3C there is net growth of *B. cereus* for disinfectant concentrations below 1.39 mg/L and the bacteriostatic effect is greater at low concentrations of DDAC whereas the bactericidal effect is higher at larger concentrations. On the other hand Figure 3D shows a similar trend, but with less bacteriostatic effect and with a MIC of 2.86 mg/L.

## 4. Conclusion

A new mathematical model capable to reproduce net growth and death kinetics is derived to study DDAC effects at sub-MIC concentrations. The model describes DDAC influence on adaptation, growth, stationary, and death phases and discerns if the disinfectant is bactericidal, bacteriostatic, or both, and at which concentrations. The model also considers adaptation, which is relevant to detect if the absence of growth after treatment is due to the use of supra-MIC treatments or because there is a strong delay in the lag phase and bacteria can grow hours later, as observed in *E. coli* experiments. The analysis of the model reveals that DDAC is both bacteriostatic and bactericidal but mainly bacteriostatic at low sub-MIC concentrations and bactericidal at large sub-MIC concentrations.

Moreover, the model is able to reproduce the behavior of both OD and CFUs measurements using the same mechanisms and it is sufficiently flexible to account for different forms of the disinfectant effect and could be used to study the mechanisms of action of different types of antimicrobials, like antibiotics, at sub-MIC concentrations.

## Data Availability Statement

The datasets and mathematical models presented in this study can be found online in the following public repository https://doi.org/10.5281/zenodo.5167910.

## Author Contributions

AP and JAV designed the experimental study, conducted the experiments, and analyzed the data. AP and MG designed the theoretical study and conducted the computational experiments and analysis. All the authors drafted the manuscript and approved the final version.

## Funding

This study was funded by the projects RTI2018-093560-J-I00 (MCIU/AEI/FEDER, UE), RYC2019-028006-I/AIE/10.13039/501100011033, 20213AT001, and the Xunta de Galicia Grants IN606A-2020/028 and IN607B-2021/11.

## Conflict of Interest

The authors declare that the research was conducted in the absence of any commercial or financial relationships that could be construed as a potential conflict of interest.

## Publisher's Note

All claims expressed in this article are solely those of the authors and do not necessarily represent those of their affiliated organizations, or those of the publisher, the editors and the reviewers. Any product that may be evaluated in this article, or claim that may be made by its manufacturer, is not guaranteed or endorsed by the publisher.

## Supplementary Material

Figure S1 Experimental data showing OD and CFUs growth of *B. cereus* **(A,B)** and OD and CFUs growth of *E. coli* **(C,D)** at different concentrations of DDAC. Standard deviation (error bars) and mean value (center of error bars) are represented. Shows experimental data means and variability for *B. cereus* and *E. coli* . Model files, experimental data, and scripts to reproduce results can be found in the following public repository https://doi.org/10.5281/zenodo.5167910.

## References

[B1] Araújo P. A. Lemos M. Mergulhão F. Melo L. Simões M. (2013). The influence of interfering substances on the antimicrobial activity of selected quaternary ammonium compounds. Int. J. Food Sci. 2013, 237581. 10.1155/2013/237581 26904590 PMC4745498

[B2] Balsa-Canto E. Alonso A. Arias-Méndez A. García M. López-Núñez A. Mosquera-Fernández M.. (2016a). Modeling and Optimization Techniques With Applications in Food Processes, Bio-Processes and Bio-Systems, Vol. 9. Springer. Available online at: https://link.springer.com/chapter/10.1007/978-3-319-32146-2_4

[B3] Balsa-Canto E. Henriques D. Gábor A. Banga J. R. (2016b). AMIGO2, a toolbox for dynamic modeling, optimization and control in systems biology. Bioinformatics 32, 3357–3359. 10.1093/bioinformatics/btw411 27378288 PMC5079478

[B4] Buffet-Bataillon S. Tattevin P. Bonnaure-Mallet M. Jolivet-Gougeon A. (2012). Emergence of resistance to antibacterial agents: the role of quaternary ammonium compounds - a critical review. Int. J. Antimicrob. Agents 39, 381–389. 10.1016/j.ijantimicag.2012.01.011 22421329 10.1016/j.ijantimicag.2012.01.011

[B5] Capita R. Alonso-Calleja C. (2013). Antibiotic-resistant bacteria: a challenge for the food industry. Crit. Rev. Food Sci. Nutr. 53, 11–48. 10.1080/10408398.2010.519837 23035919

[B6] Capita R. Vicente-Velasco M. Rodríguez-Melcón C. García-Fernández C. Carballo J. Alonso-Calleja C. (2019). Effect of low doses of biocides on the antimicrobial resistance and the biofilms of Cronobacter sakazakii and Yersinia enterocolitica. Sci. Rep. 9, 1–12. 10.1038/s41598-019-51907-1 31685860 PMC6828698

[B7] Chatterjee T. Chatterjee B. K. Majumdar D. Chakrabarti P. (2015). Antibacterial effect of silver nanoparticles and the modeling of bacterial growth kinetics using a modified gompertz model. Biochimica et Biophysica Acta (BBA)-General Subjects 1850, 299–306. 10.1016/j.bbagen.2014.10.022 25450183

[B8] Coates J. Park B. R. Le D. Şimşek E. Chaudhry W. Kim M. (2018). Antibiotic-induced population fluctuations and stochastic clearance of bacteria. eLife 7, 1–26. 10.7554/eLife.32976 29508699 PMC5847335

[B9] EN-1276. (2009). Chemical disinfectants and antiseptics in: Quantitative suspension test for the evaluation of bactericidal activity of chemical disinfectants and antiseptics used in food, industrial, domestic, and institutional areas - Test method and requirements (phase 2, step 1). European Standard EN-1276, 2009. Available online at: https://standards.iteh.ai/catalog/standards/cen/d7ba84f4-a83b-46a7-8065-c638f57a9111/en-1276-2009

[B10] EURL-SRM (2016). Analysis of quaternary ammonium compounds (QACs) in fruits and vegetables using QuEChERS and LC-MS/MS. EU Ref. Lab. Residues Pesticides Single Residue Methods 5, 1–6. Available online at: https://www.eurl-pesticides.eu/userfiles/file/EurlSRM/EurlSRM_meth_QAC_ShortMethod.pdf

[B11] Fykse E. M. Olsen J. S. Skogan G. (2003). Application of sonication to release dna from bacillus cereus for quantitative detection by real-time pcr. J. Microbiol. Methods 55, 1–10. 10.1016/S0167-7012(03)00091-5 14499990

[B12] García M. R. Cabo M. L. (2018). Optimization of E. coli inactivation by benzalkonium chloride reveals the importance of quantifying the inoculum effect on chemical disinfection. Front. Microbiol. 9, 1259. 10.3389/fmicb.2018.01259 29997577 PMC6028699

[B13] Gyrk L. L. Finch G. R. (1998). Modeling water treatment chemical disinfection kinetics. J. Environ. Eng. 124, 783–793.

[B14] Haque M. A. Imamura R. Brown G. A. Krishnamurthi V. R. Niyonshuti I. I. Marcelle T.. (2017). An experiment-based model quantifying antimicrobial activity of silver nanoparticles on: escherichia coli. RSC Adv. 7, 56173–56182. 10.1039/C7RA10495B

[B15] Holah J. Taylor J. Dawson D. Hall K. (2002). Biocide use in the food industry and the disinfectant resistance of persistent strains of Listeria monocytogenes and Escherichia coli. J. Appl. Microbiol. 92, 111S–120S. 10.1046/j.1365-2672.92.5s1.18.x 12000620

[B16] ISO-20776-1. (2019). Susceptibility testing of infectious agents and evaluation of performance of antimicrobial susceptibility test devices - Part 1: Broth micro-dilution reference method for testing the in vitro activity of antimicrobial agents against rapidly growing aerobic bacteria involved in infectious diseases. ISO 20776-1, 2019. Available online at: https://www.iso.org/standard/70464.html

[B17] Kampf G.. (2018a). Antiseptic Stewardship. Springer. Available online at: https://link.springer.com/content/pdf/10.1007/978-3-319-98785-9.pdf

[B18] Kampf G.. (2018b). Biocidal agents used for disinfection can enhance antibiotic resistance in gram-negative species. Antibiotics 7, 110. 10.3390/antibiotics7040110 30558235 PMC6316403

[B19] Lambert R. J. Pearson J. (2000). Susceptibility testing: accurate and reproducible minimum inhibitory concentration (MIC) and non-inhibitory concentration (NIC) values. J. Appl. Microbiol. 88, 784–790. 10.1046/j.1365-2672.2000.01017.x 10792538

[B20] Langsrud S. Sundheim G. Borgmann-Strahsen R. (2003). Intrinsic and acquired resistance to quaternary ammonium compounds in food-related Pseudomonas spp. J. Appl. Microbiol. 95, 874–882. 10.1046/j.1365-2672.2003.02064.x 12969304

[B21] LeeNari Yoon K. Kyung O. ChangHyun-Joo Sook C. ChoiSung-Wook (2014). A Multiplex PCR assay for simultaneous detection of escherichia coli O157:H7, bacillus cereus, vibrio parahaemolyticus, salmonella spp., listeria monocytogenes, and staphylococcus aureus in Korean ready-to-eat food. Foodborne Pathog Dis. 11, 574–580. 10.1089/fpd.2013.1638 24796416

[B22] Lim C. H. Chung Y. H. (2014). Effects of didecyldimethylammonium chloride on sprague-dawley rats after two weeks of inhalation exposure. Toxicol. Res. 30, 205–210. 10.5487/TR.2014.30.3.205 25343015 PMC4206748

[B23] Liu P. Rand K. H. Obermann B. Derendorf H. (2005). Pharmacokinetic-pharmacodynamic modelling of antibacterial activity of cefpodoxime and cefixime in in vitro kinetic models. Int. J. Antimicrob. Agents 25, 120–129. 10.1016/j.ijantimicag.2004.09.012 15664481

[B24] Mouton J. W. Vinks A. A. (2005). Pharmacokinetic/pharmacodynamic modelling of antibacterials in vitro and in vivo using bacterial growth and kill kinetics the minimum inhibitory concentration versus stationary concentration. Clin. Pharmacokinet. 44, 201–210. 10.2165/00003088-200544020-00005 15656698

[B25] Nordholt N. Kanaris O. Schmidt S. B. Schreiber F. (2021). Persistence against benzalkonium chloride promotes rapid evolution of tolerance during periodic disinfection. Nat. Commun. 12, 1–13. 10.1038/s41467-021-27019-8 34815390 PMC8611074

[B26] Pedreira A. Taşkn Y. García M. R. (2021). A critical review of disinfection processes to control SARS-CoV-2 transmission in the food industry. Foods 10, 283. 10.3390/foods10020283 33572531 PMC7911259

[B27] Peleg M.. (2021). Modeling the dynamic kinetics of microbial disinfection with dissipating chemical agents–a theoretical investigation. Appl. Microbiol. Biotechnol. 105, 539–549. 10.1007/s00253-020-11042-8 33394150 PMC7780086

[B28] Ramzi A. Oumokhtar B. Ez Zoubi Y. Filali Mouatassem T. Benboubker M. El Ouali Lalami A. (2020). Evaluation of antibacterial activity of three quaternary ammonium disinfectants on different germs isolated from the hospital environment. BioMed Res. Int. 2020, 6509740. 10.1155/2020/6509740 33381566 PMC7749782

[B29] Regoes R. R. Wiuff C. Zappala R. M. Garner K. N. Baquero F. Levin B. R. (2004). Pharmacodynamic functions: a multiparameter approach to the design of antibiotic treatment regimens. Antimicrob. Agents Chemother. 48, 3670. 10.1128/AAC.48.10.3670-3676.2004 15388418 PMC521919

[B30] Rial D. Vázquez J. A. Murado M. A. (2011). Effects of three heavy metals on the bacteria growth kinetics: a bivariate model for toxicological assessment. Appl. Microbiol. Biotechnol. 90, 1095–1109. 10.1007/s00253-011-3138-1 21360150

[B31] Ribič U. Jakše J. Toplak N. Koren S. Kovač M. Klančnik A.. (2020). Transporters and efflux pumps are the main mechanisms involved in staphylococcus epidermidis adaptation and tolerance to didecyldimethylammonium chloride. Microorganisms 8, 344. 10.3390/microorganisms8030344 32121333 PMC7143832

[B32] Santillán M.. (2008). On the use of the hill functions in mathematical models of gene regulatory networks. Math. Model. Nat. Phenom 3, 85–97. 10.1051/mmnp:2008056

[B33] Simões M. Simões L. C. Vieira M. J. (2010). A review of current and emergent biofilm control strategies. LWT Food Sci. Technol. 43, 573–583. 10.1016/j.lwt.2009.12.008

[B34] Soumet C. Méheust D. Pissavin C. Le Grandois P. Frémaux B. Feurer C.. (2016). Reduced susceptibilities to biocides and resistance to antibiotics in food-associated bacteria following exposure to quaternary ammonium compounds. J. Appl. Microbiol. 121, 1275–1281. 10.1111/jam.13247 27481186

[B35] Stevenson K. McVey A. F. Clark I. B. N. Swain P. S. Pilizota T. (2016). General calibration of microbial growth in microplate readers. Sci. Rep. 6, 1–7. 10.1038/srep38828 27958314 PMC5153849

[B36] Theophel K. Schacht V. J. Schlüter M. Schnell S. Stingu C. S. Schaumann R.. (2014). The importance of growth kinetic analysis in determining bacterial susceptibility against antibiotics and silver nanoparticles. Front. Microbiol. 5, 544. 10.3389/fmicb.2014.00544 25426104 PMC4226228

[B37] Vázquez J. A. Durán A. Rodríguez-Amado I. Prieto M. A. Rial D. Murado M. A. (2011). Evaluation of toxic effects of several carboxylic acids on bacterial growth by toxicodynamic modelling. Microb. Cell Fact. 10, 100. 10.1186/1475-2859-10-100 22118421 PMC3235065

[B38] Walsh S. E. Maillard J. Y. Russell A. D. Catrenich C. E. Charbonneau D. L. Bartolo R. G. (2003). Activity and mechanisms of action of selected biocidal agents on Gram-positive and -negative bacteria. J. Appl. Microbiol. 94, 240–247. 10.1046/j.1365-2672.2003.01825.x 12534815

[B39] Yoshimatsu T. Hiyama K. I. (2007). Mechanism of the action of didecyldimethylammonium chloride (DDAC) against Escherichia coli and morphological changes of the cells. Biocontrol Sci. 12, 93–99. 10.4265/bio.12.93 17927049

[B40] Zhang A. He X. Meng Y. Guo L. Long M. Yu H.. (2016). Antibiotic and disinfectant resistance of escherichia coli isolated from retail meats in Sichuan, China. Microb. Drug Resist. 22, 80–87. 10.1089/mdr.2015.0061 26167743

[B41] Zhang C. Cui F. Zeng G. m. Jiang M. Yang Z. Z. Yu Z. G.. (2015). Quaternary ammonium compounds (QACs): a review on occurrence, fate and toxicity in the environment. Sci. Total Environ. 518–519, 352–362. 10.1016/j.scitotenv.2015.03.007 25770948

[B42] Zou L. Meng J. McDermott P. F. Wang F. Yang Q. Cao G.. (2014). Presence of disinfectant resistance genes in Escherichia coli isolated from retail meats in the USA. J. Antimicrob. Chemother. 69, 2644–2649. 10.1093/jac/dku197 24908046

