# Erratum: Kinetics of bacterial adaptation, growth, and death at didecyldimethylammonium chloride sub-MIC concentrations

**DOI:** 10.3389/fmicb.2023.1187751

**Published:** 2023-03-24

**Authors:** Frontiers Production Office

**Affiliations:** Frontiers Media SA, Lausanne, Switzerland

**Keywords:** dynamic modeling, disinfection, didecyldimethylammonium chloride (DDAC), *B. cereus*, *E. coli*, bacteriostatic, bactericidal, sub-MIC concentration

Due to a production error, there was a mistake in Figure 1 as published. The image published as Figure 1 in the downloadable PDF was incorrect. The corrected Figure 1 appears below.

**Figure 1:**
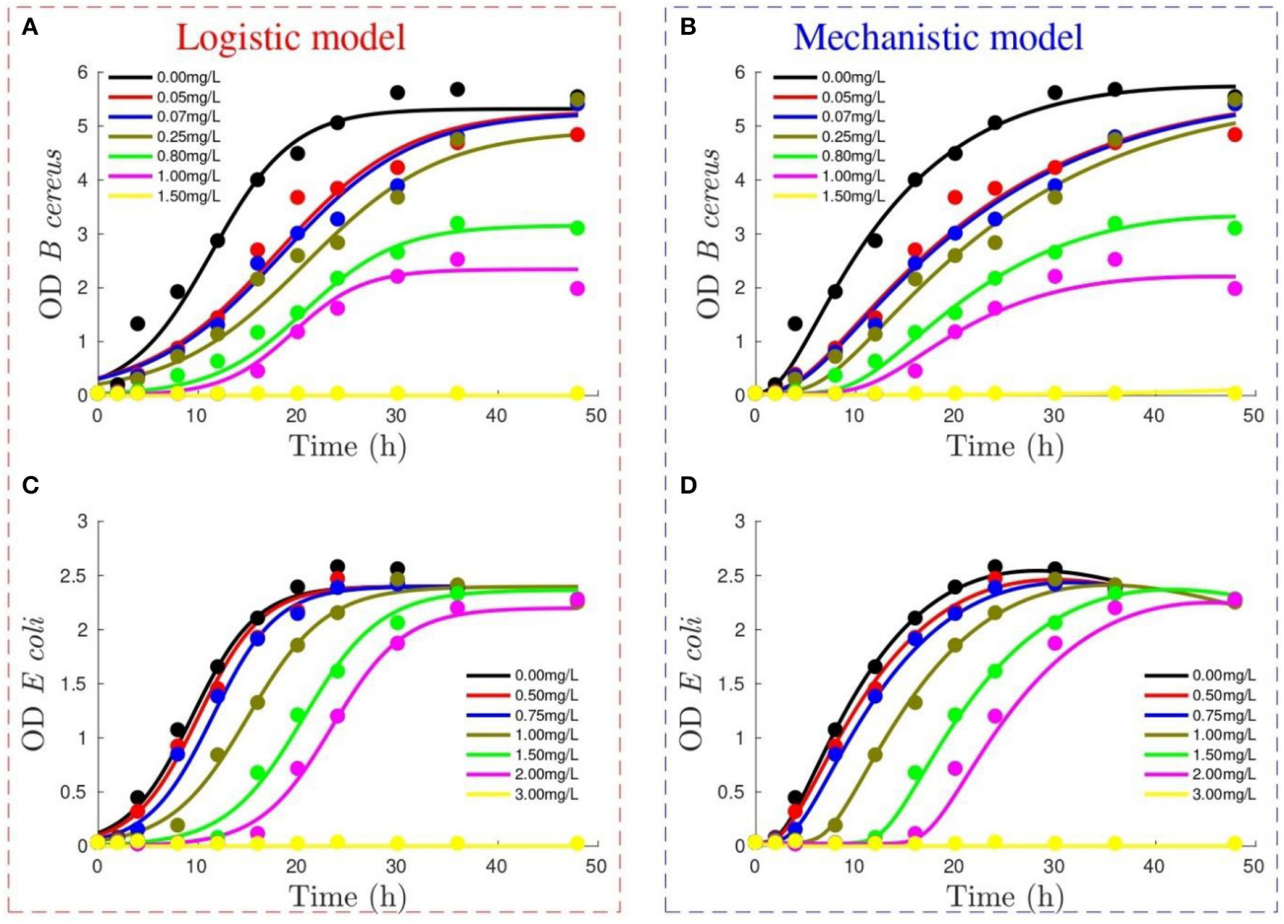
Performance of logistic model (figures on the left) and mechanistic model (figures on the right) to reproduce optical density (OD) growth of *Bacillus cereus* **(A,B)** and *Escherichia coli* **(C,D)** at different Didecyldimethylammonium chloride (DDAC) concentrations (refer to legend). Lines show model output, whereas experimental data are represented by dots.

The publisher apologizes for this mistake. The original article has been updated.

