# Supplementary figures and images for: Kinetics of Bacterial Adaptation, Growth, and Death at Didecyldimethylammonium Chloride sub-MIC Concentrations
Source: Front Microbiol. 2022 Apr 7;13:758237. doi: 10.3389/fmicb.2022.758237 (PMC9023358; doi:10.3389/fmicb.2022.758237)

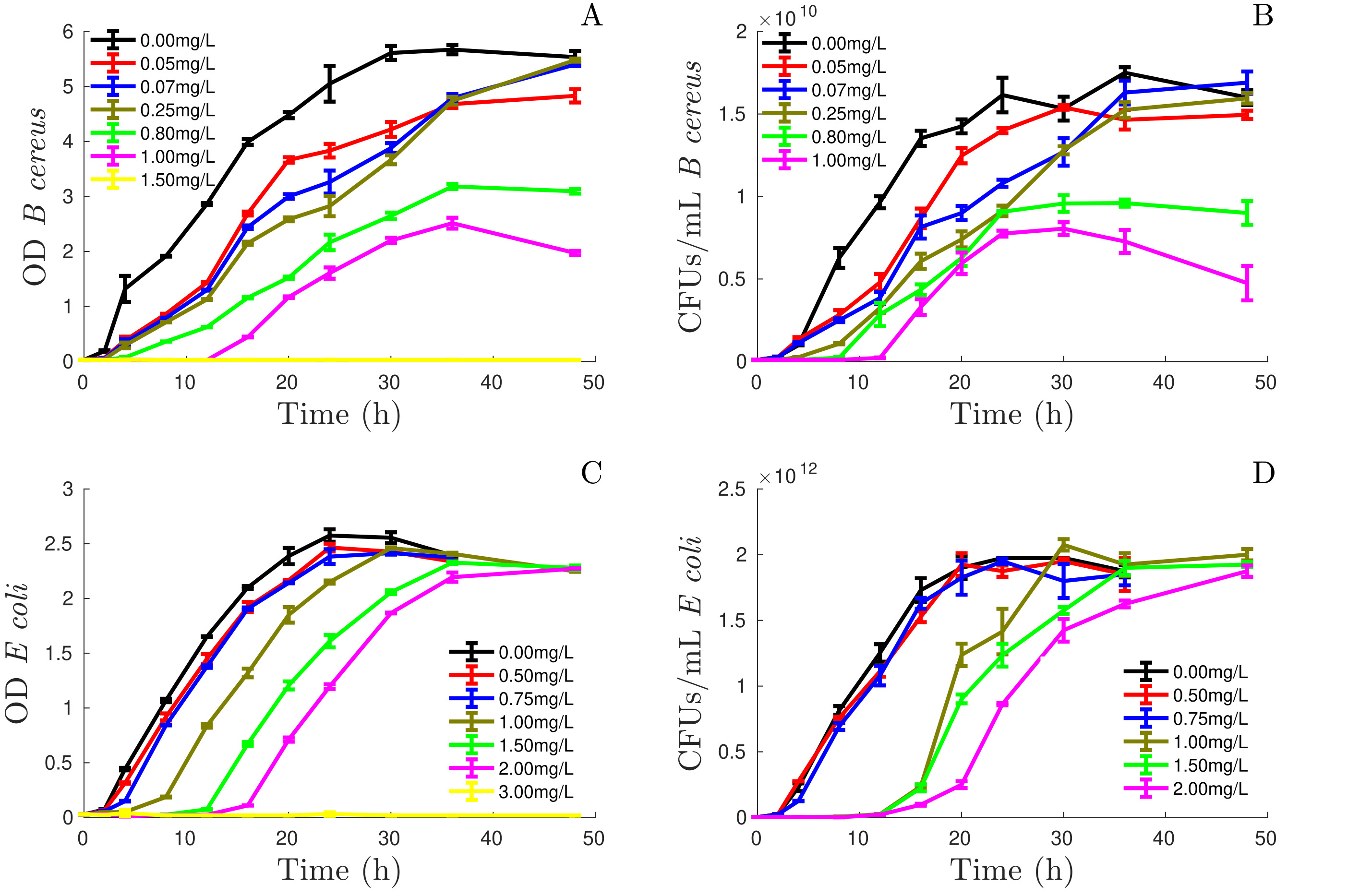

Supplement: Figure S1 — Experimental data showing OD and CFUs growth of B. cereus (A,B) and OD and CFUs growth of E. coli (C,D) at different concentrations of DDAC. Standard deviation (error bars) and mean value (center of error bars) are represented. Shows experimental data means and variability for B. cereus and E. coli . Model files, experimental data, and scripts to reproduce results can be found in the following public repository https://doi.org/10.5281/zenodo.5167910. [file Image_1.jpeg]
